# Identification and validation of a TTN-associated immune prognostic model for skin cutaneous melanoma

**DOI:** 10.3389/fgene.2022.1084937

**Published:** 2023-01-10

**Authors:** Qirui Wang, Xingtai Huang, Siyi Zeng, Renpeng Zhou, Danru Wang

**Affiliations:** ^1^ Department of Plastic and Reconstructive Surgery, Shanghai Ninth People’s Hospital, Shanghai Jiao Tong University School of Medicine, Shanghai, China; ^2^ Shanghai Key Laboratory of Stomatology, National Center for Stomatology, National Clinical Research Center for Oral Diseases, Department of Orthodontics, College of Stomatology, Shanghai Ninth People’s Hospital, Shanghai Jiao Tong University School of Medicine, Shanghai, China

**Keywords:** skin cutaneous melanoma (SKCM), TTN, mutation, immune prognostic model, prognosis

## Abstract

TTN is the most commonly mutated gene in skin cutaneous melanoma (SKCM). Tumor mutational burden (TMB) can generate new antigens that regulate the recognition of T cells, which will significantly affect the prognosis of patients. The TTN gene has a long coding sequence and a high number of mutant sites, which allows SKCM patients to produce higher TMB and may influence the immune response. It has been found that the overall survival (OS) of SKCM patients with TTN mutation was significantly higher than that of wild-type patients. However, the effect of TTN mutation on the immune microenvironment of SKCM has not been fully investigated. Here, we systematically explored the relationship and potential mechanisms between TTN mutation status and the immune response. We first revealed that TTN mutated SKCM were significantly associated with four immune-related biological processes. Next, 115 immune genes differentially expressed between TTN mutation and wild-type SKCM patients were found to significantly affect the OS of SKCM patients. Then, we screened four immune-related genes (CXCL9, PSMB9, CD274, and FCGR2A) using LASSO regression analysis and constructed a TTN mutation-associated immune prognostic model (TM-IPM) to distinguish the SKCM patients with a high or low risk of poor prognosis, independent of multiple clinical characteristics. SKCM in the low-risk group highly expressed a large number of immune-related genes, and functional enrichment analysis of these genes showed that this group was involved in multiple immune processes and pathways. Furthermore, the nomogram constructed by TM-IPM with other clinicopathological parameters can provide a predictive tool for clinicians. Moreover, we found that CD8^+^ T cells were significantly enriched in the low-risk group. The expression level of immune checkpoints was higher in the low-risk group than in the high-risk group. Additionally, the response to chemotherapeutic agents was higher in the low-risk group than in the high-risk group, which may be related to the long survival in the low-risk group. Collectively, we constructed and validated a TM-IPM using four immune-related genes and analyzed the potential mechanisms of TM-IPM to predict patient prognosis and response to immunotherapy from an immunological perspective.

## Introduction

Melanoma is a type of cancer caused by malignant lesions of melanocytes. Furthermore, skin cutaneous melanoma (SKCM) is the most common subtype of melanoma, comprising over 90% of all melanomas (Ali et al., 2013) and ranking 15th among most common malignancy worldwide (Leonardi et al., 2018). With the exception of non-melanoma skin cancer, newly diagnosed SKCM accounts for approximately 1.7% of all primary malignancies in the world (Schadendorf et al., 2018). Recent studies have found that melanoma is very sensitive to immunomodulation and is gradually becoming a major treatment (Weiss et al., 2019). However, immunotherapy is only available for a subset of the population, which is usually high in the expression of immune checkpoints. The introduction of immune checkpoint inhibitors (ICIs) targeting immune checkpoints such as cytotoxic T-lymphocyte antigen-4 (CTLA-4) and programmed death 1 (PD-1) has significantly improved and extended the life span of patients (Weiss et al., 2019). Therefore, it is crucial to develop an immune prognostic model that can accurately predict whether SKCM patients will respond to immunotherapy and their prognosis.

It is reported that about 65% of SKCM is caused by ultraviolet radiation (UVR), which can cause DNA alteration (Emri et al., 2018). Importantly, the gene with the highest mutation frequency in SKCM is titin (TTN) (Kang et al., 2020). TTN is used to express myosin, which is responsible for maintaining muscle tension (Savarese et al., 2018). The TTN gene has a long coding sequence and mutations at any site may lead to abnormal myosin function, which will result in aberrant growth of muscle fibers (Savarese et al., 2018). Numerous previous studies have found that TTN mutation is associated with diseases such as tibial muscular dystrophy (Hackman et al., 2002), moyamoya disease (Xiao et al., 2022) and familial atrioventricular block (Liu et al., 2020). However, recently scholars have focused on the relationship between TTN mutation and immunotherapy for solid tumors (Miao et al., 2018; Jia et al., 2019).

Recently, one study demonstrated that TTN mutation in lung squamous carcinoma (LUSC) was positively associated with tumor mutational burden (TMB) and served as an independent prognostic factor and influenced the immune microenvironment of LUSC (Zou et al., 2022). Higher TMB produces more neoantigens, which increases the chance of recognition by T cells (Jardim et al., 2021). Interestingly, in SKCM, TMB was positively correlated with prognosis and response to immunotherapy. The TTN gene, which has a high number of mutant loci, is an essential component of TMB. However, the role of TTN mutation in SKCM has not been clearly elucidated. In this study, based on The Cancer Genome Atlas (TCGA) database, we compared immune-related biological processes between TTN mutated and wild-type SKCM and obtained immune-related genes. In addition, we constructed and validated a TTN mutation-associated immune prognostic model (TM-IPM) based on immune-associated genes and developed a nomogram with clinical characteristics. In general, the results of this study will provide a novel risk prediction model for the diagnosis, prognosis and treatment of SKCM from an immune perspective.

## Materials and methods

### Data source and mutation analysis

Transcriptome data (RNA-sequencing [RNA-seq] data, FPKM format), genetic mutation and clinical data for 471 SKCM patients were downloaded from TCGA (http://cancergenome.nih.gov) database. Following the previous method of Bo Li et al. (Li et al., 2010), we converted the FPKM format of the expression spectrum from TCGA database to TPM format. Mutation data were analyzed using the *maftools* package in R software. The histogram showed the top 10 genes with the highest mutation frequency in SKCM patients. Furthermore, we obtained 367 TTN mutated samples and 98 TTN non-mutated samples from the TCGA database, which served as the training cohort.

In addition, gene expression profile matrix files and clinical information of GSE65904 (including 214 melanoma samples) based on the GPL10558 platform were downloaded from the Gene Expression Omnibus (GEO, https://www.ncbi.nlm.nih.gov/geo/) database. For the expression matrix of the above data, we removed genes and samples with NA values greater than 50% and used the *impute.knn* function of the *impute* package to perform missing value completion, setting number of neighbors to 10 to complete the missing data. Moreover, we performed a log2 (X+1) transformation on the microarray data of GSE65904. It was then normalized using the *normalizeBetweenArrays* function of the *limma* package. Further, Q-Q plots were plotted to verify the normalized data (Figure S1). The data of GSE65904 served as the validation cohort. The data for this study were obtained from public databases, which do not require ethical approval and informed consent.

### Gene set enrichment analysis (GSEA)

To determine the differences in immune pathways and associated immune genes between SKCM samples with (*n* = 367) and without (*n* = 98) TTN mutation in the TCGA SKCM cohort, we performed GSEA analysis. For the GSEA analysis, we used the R package *clusterProfiler*. We selected c5.bp.v6.2.symbols.gm as the reference gene set, which was downloaded from Molecular Signatures Database (http://www.gsea-msigdb.org/gsea/downloads.jsp) (Liberzon et al., 2011) to assess relevant biological functions. The minimum gene set was set at five and the maximum gene set at 5,000, and the sampling was repeated one thousand times. *p*-value < 0.05 and false discovery rate (FDR) < 0.25 were considered statistically significant. According to the GSEA results, we obtained 115 key immune-related genes (IRGs).

### Construction and validation of an immune-related prognostic model

In this study, we evaluated the prognostic significance of each key genes using the Cox method by the *survival* package. Then, we identified prognostic risk characteristics using the Least Absolute Shrinkage and Selection Operator (LASSO) regression method (Gui and Li, 2005). LASSO regression was performed by using the *glmnet* package of R software, which was used to integrate survival time, survival status and gene expression data. Further, we set up a 10-fold cross validation to obtain the optimal model. Then, the final four genes were identified for the construction of our signature. The risk score was calculated using the following formula: Risk Score = $\overset{i=1}{\sum }\left({coef}_{i}*{Expr}_{i}\right)$ , where *coef*_*i*_ is the coefficient and *Expr*_*i*_ is the related expression value. The 214 samples in GSE65904 were used to validate this prognostic risk model. Subsequently, we evaluated the performance of the prognostic model. The *maxstat* package was used to find the optimal cutoff point for SKCM patients classified as low- and high-risk groups. Further, we analyzed the prognostic differences between the two groups using the *survival* package, and assessed the significance of prognostic differences between the samples of different groups using the log-rank test method. The overall survival (OS) of the patients is presented by the Kaplan-Meier (K-M) curves. Moreover, we plotted receiver operating characteristic (ROC) curves by the *pROC* package.

### Identification of differentially expressed genes and immune-related genes

The *limma* package (version 3.40.6) of R software was used to perform differential analysis to obtain differential expressed genes (DEGs) between low- and high-risk groups. Absolute fold change ≥3 and adjusted *p*-value < 0.05 were considered significant. The volcano plots and heatmaps were drawn in R using the *ggplot2* and *pheatmap* package, respectively. Using Euclidean distance metric and complete agglomeration method, hierarchical clusters were generated. Next, we downloaded the immune-related gene list from the Immport website (https://www.immport.org/home). By taking the intersection of DEGs with immune-related genes, we obtained the differential immune-related genes (IRGs). This was shown with a Venn diagram.

### Functional enrichment analysis

According to the DEGs obtained in the previous step, we performed Gene Ontology (GO) and Kyoto Encyclopedia of Genes and Genomes (KEGG) pathway analyses for upregulated genes (high-risk group) and downregulated genes (low-risk group), respectively. For the GO and KEGG analysis, we used the R package *clusterProfiler* and *org.Hs.eg.db*.

### Univariate and multivariate Cox regression analysis

The 355 SKCM samples with survival information from the TCGA database contained various clinical features, including age, sex, T/N/M stage, and pathologic stage. To verify whether the prognostic model predictions were independent of traditional clinical characteristics (including age, sex, T/N/M stage, and pathologic stage), we performed univariate and multivariate Cox regression analyses on SKCM patients.

### Construction and evaluation of nomogram and decision curve analysis (DCA)

Based on the results of the multivariate analysis, a nomogram was constructed to estimate the predicted survival probability for 1 year, 3 years, and 5 years. The *rms* package (version 6.2–0) was used to build the nomogram, which was calibrated using calibration plots. Moreover, the nomogram’s performance was assessed using the concordance index (C-index). Furthermore, the DCA was used to evaluate the clinical benefits of the nomogram over a single prognostic factor.

### Immune infiltration analysis

From gene expression data, the 64 immune and stromal cell types in SKCM tumors were evaluated by the xCell, CIBERSORTx, quanTIseq and ESTIMATE algorithms (Aran et al., 2017; Rusk, 2019; Plattner et al., 2020). The *estimate* package was used to assess the immune microenvironment of SKCM: the presence of stroma (Stromal Score), the level of immune cells infiltrations (Immune Score), and the sum of stromal score and immune score (Estimate Score) (Yoshihara et al., 2013). We used heat maps to show the scores of immune and stromal cells in all samples and ranked the samples using risk score. Further, we showed the scores of cell types that were significantly different in the high-risk and low-risk groups in the form of bar charts, which were statistically performed using independent samples t-tests. We analyzed the expression levels of six immune checkpoint-related genes (PDCD1, TIGIT, CD160, TIM3, CTLA4, and LAG3) in both groups, which were statistically performed using independent samples t-tests.

### Chemotherapy response prediction

Based on the public database of Genomics of Drug Sensitivity in Cancer (GDSC; https://www.cancerrxgene.org), we evaluated the half-maximal inhibitory concentration (IC50) of response to chemotherapy drugs in different risk groups using the *pRRophetic* package (Geeleher et al., 2014). We compared the difference in IC50 values between the low- and high-risk groups by Wilcoxon analysis. Additionally, the NCI-60 database is now widely used to explore the correlation between gene expression and drug sensitivity in different cancer cell lines and is available through the CellMiner database (https://discover.nci.nih.gov/cellminer) (Shankavaram et al., 2009). Pearson correlation analysis was performed to investigate potential differences in drug sensitivity between the high- and low-risk groups.

### Statistical analyses

In this study, all analyses were performed using R software (version 3.6.3) and the Sangerbox online platform (Shen et al., 2022). Pearson’s rank tests were used to explore the correlation. The specific statistical methods are described in detail in the methods above. All statistical tests were two-tailed, and *p*-value < 0.05 was considered statistically significant.

## Results

### Association of immunophenotypes with TTN mutation in SKCM

**FIGURE 1:**
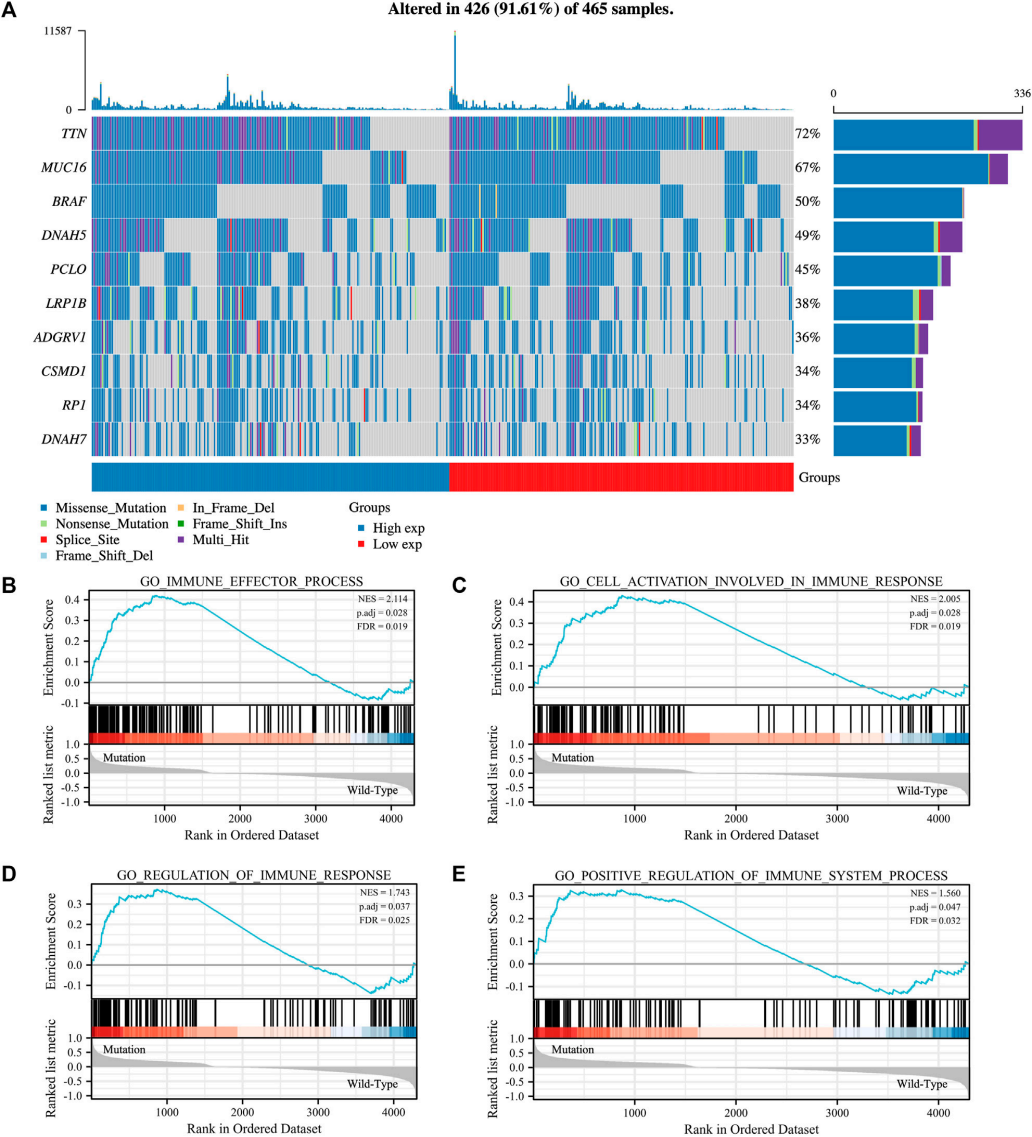
Mutated gene spectrum and Gene set enrichment analysis (GSEA) of SKCM patients based on TTN mutation status. **(A)** Mutated gene spectrum of SKCM from TCGA database (top 10). Blue represented TTN high expression group; red represented TTN low expression group. **(B–E)** GSEA between TTN mutation patients and wild-type patients. Significantly enriched immune-related biological processes between TTN mutant and wild-type comparison.

The TTN mutation is the most common type of mutation in SKCM (Figure 1A). The somatic mutation rate of TTN reached 72%. In addition, the proportion of mutations in the high and low expression groups of TTN was approximately the same. To investigate the effect of TTN mutation on SKCM, GSEA analysis was performed on SKCM with (*n* = 367) and without (*n* = 98) TTN mutation. The results showed that TTN mutant SKCM were significantly enriched in 295 biological processes (Supplementary Table S1), which contained four immune-related biological processes: GO_CELL_ACTIVATION_INVOLVED_IN_IMMUNE_RESPONSE (normalized enrichment score, NES = 2.005, p.adj = 0.028, FDR = 0.019), GO_IMMUNE_EFFECTOR_PROCESS (NES = 2.114, p.adj = 0.028, FDR = 0.019), GO_REGULATION_OF_IMMUNE_RESPONSE (NES = 1.743, p.adj = 0.037, FDR = 0.025), GO_POSITIVE_REGULATION_OF_IMMUNE_SYSTEM_PROCESS (NES = 1.560, p.adj = 0.047, FDR = 0.032) (Figures 1B–E). In contrast, TTN wild-type SKCM was not enriched for any biological processes associated with immunity.

### Construction of a TM-IPM and evaluation of its predictive capabilities in the TCGA SKCM cohort

**FIGURE 2:**
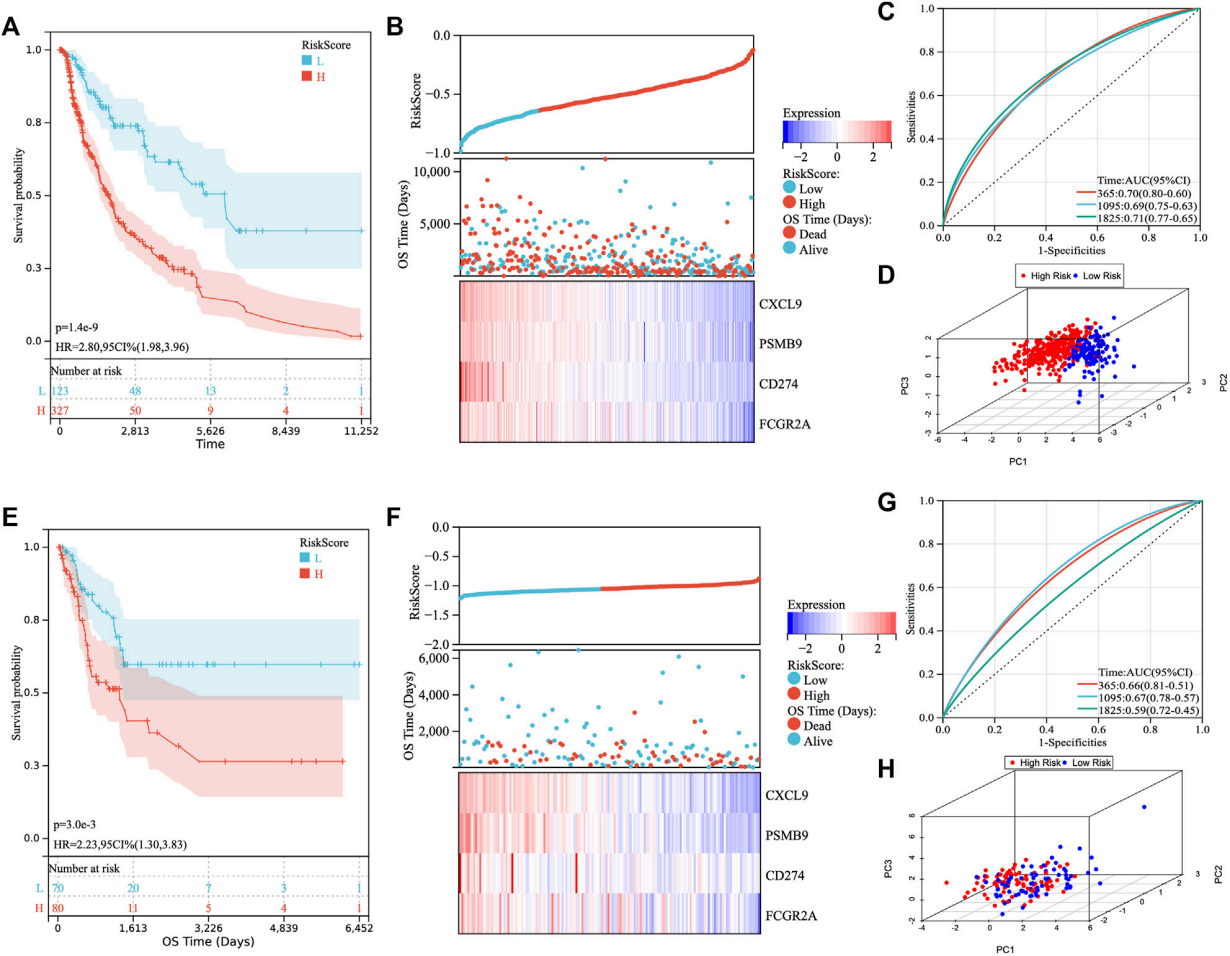
Development and validation of the TTN mutation-associated immune prognostic model (TM-IPM). Kaplan-Meier survival, risk score and time-dependent ROC curves of the TM-IPM for the TCGA SKCM cohort **(A–C)** and GSE65904 cohort **(E–G)**. TM-IPM was able to clearly classify SKCM patients from TCGA **(D)** and GEO **(H)** into high and low-risk groups.

We obtained 115 key IRGs from the four immune-related biological processes enriched by GSEA (Supplementary Table S2). To identify immune genes associated with prognosis, we first performed univariate Cox regression analysis on IRGs and obtained a total of 48 genes with prognostic value (Supplementary Figure S2A). Next, we applied LASSO regression analysis to identify the genes with the greatest prognostic value. Finally, the LASSO regression analysis incorporated four genes (CXCL9, PSMB9, CD274, and FCGR2A) for constructing the prediction model (Supplementary Figures S2B, C). Then, the LASSO regression coefficients were multiplied by the expression value of each immune gene to construct a risk score model (risk score = -0.028*CXCL9-0.009*PSMB9-0.045*CD274-0.048*FCGR2A). We divided the patients into high- and low-risk groups based on risk scores (Figure 2D). There was a shorter OS for high-risk patients compared with low-risk patients in TCGA cohort (Figure 2A). Figure 2B illustrated the distribution of risk scores and gene expression data. Time-dependent ROC curves were used to illustrate the predictive power of the TM-IPM (Figure 2C). In the prognostic model for OS, the area under the ROC curve (AUC) was 0.70 at 1 year, 0.69 at 3 years, and 0.71 at 5 years.

### Validation and evaluation of the TM-IPM in the GEO SKCM cohort

**FIGURE 3:**
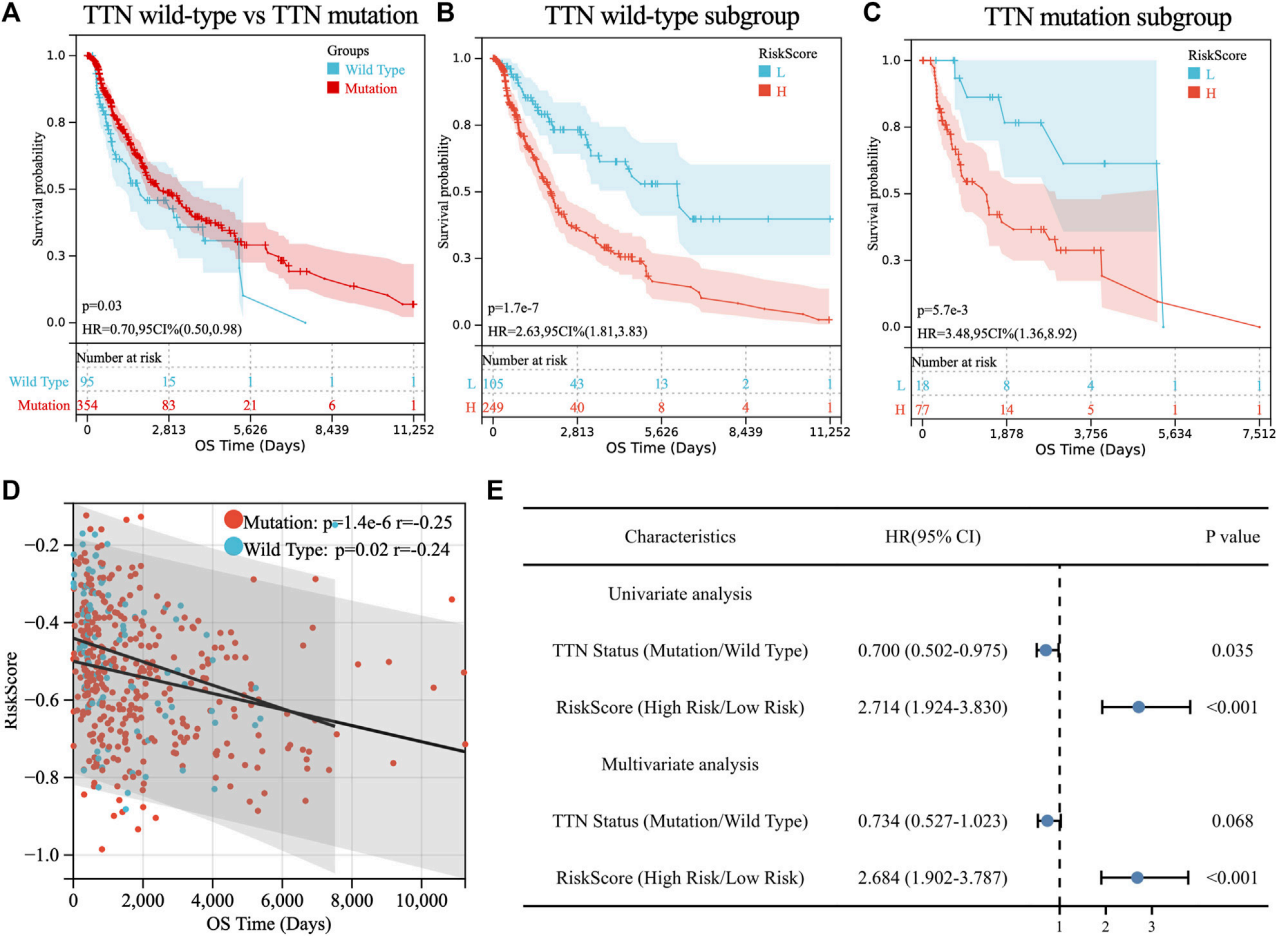
Prognostic analysis of TTN mutation in SKCM patients. **(A)** Kaplan-Meier survival between TTN mutation and wild-type patients. **(B)** Kaplan-Meier survival between high and low-risk groups in TTN wild-type patients. **(C)** Kaplan-Meier survival between high and low-risk groups in TTN mutation patients. **(D)** Correlation between riskscore and overall survival (OS) in SKCM patients with TTN mutation and wild type. **(E)** Univariate and multivariate regression analysis of TTN status and riskscore of TM-IPM.

To determine whether TM-IPM is stable, the performance of TM-IPM was evaluated in a GEO SKCM cohort consisting of 214 SKCM patients. Patients in the GEO SKCM cohort were calculated using the same formula for risk score and categorized into high-risk and low-risk groups (Figure 2E). Consistent with the results of the TCGA SKCM cohort, patients in the low-risk group had significantly higher OS than those in the high-risk group (Figure 2F). Furthermore, Figure 3B showed the distribution of risk scores and gene expression data. The AUC was 0.66 at 1 year, 0.67 at 3 years, and 0.59 at 5 years (Figure 2G).

### Stratification analyses of OS based on TTN status for the TM-IPM in the TCGA SKCM cohort

Consistent with TM-IPM, TTN status was also significantly associated with the prognosis of SKCM patients. However, interestingly, patients with TTN mutation had higher OS than those with TTN wild type (Figure 3A). Next, to test whether the prognostic value of TM-IPM was independent of TTN status, stratification analyses were performed. Thus, patients in the TCGA SKCM cohort were divided into TTN mutation subgroup and TTN wild-type subgroup based on TTN status. Based on stratification analyses, both the TTN wild-type and TTN mutation subgroups showed significant relationships between TM-IPM and OS. Low-risk patients in both subgroups had a better prognosis (Figures 3B,C). Moreover, correlation analysis showed that risk score was significantly negatively associated with OS time in both subgroups of the TCGA SKCM cohort (Figure 3D). In addition, univariate and multivariate Cox regression analyses showed that TM-IPM could be an independent predictor of OS in SKCM patients and independent of TTN status (Figure 3E).

### Low risk predicted an enhanced local immune infiltration in SKCM

**FIGURE 4:**
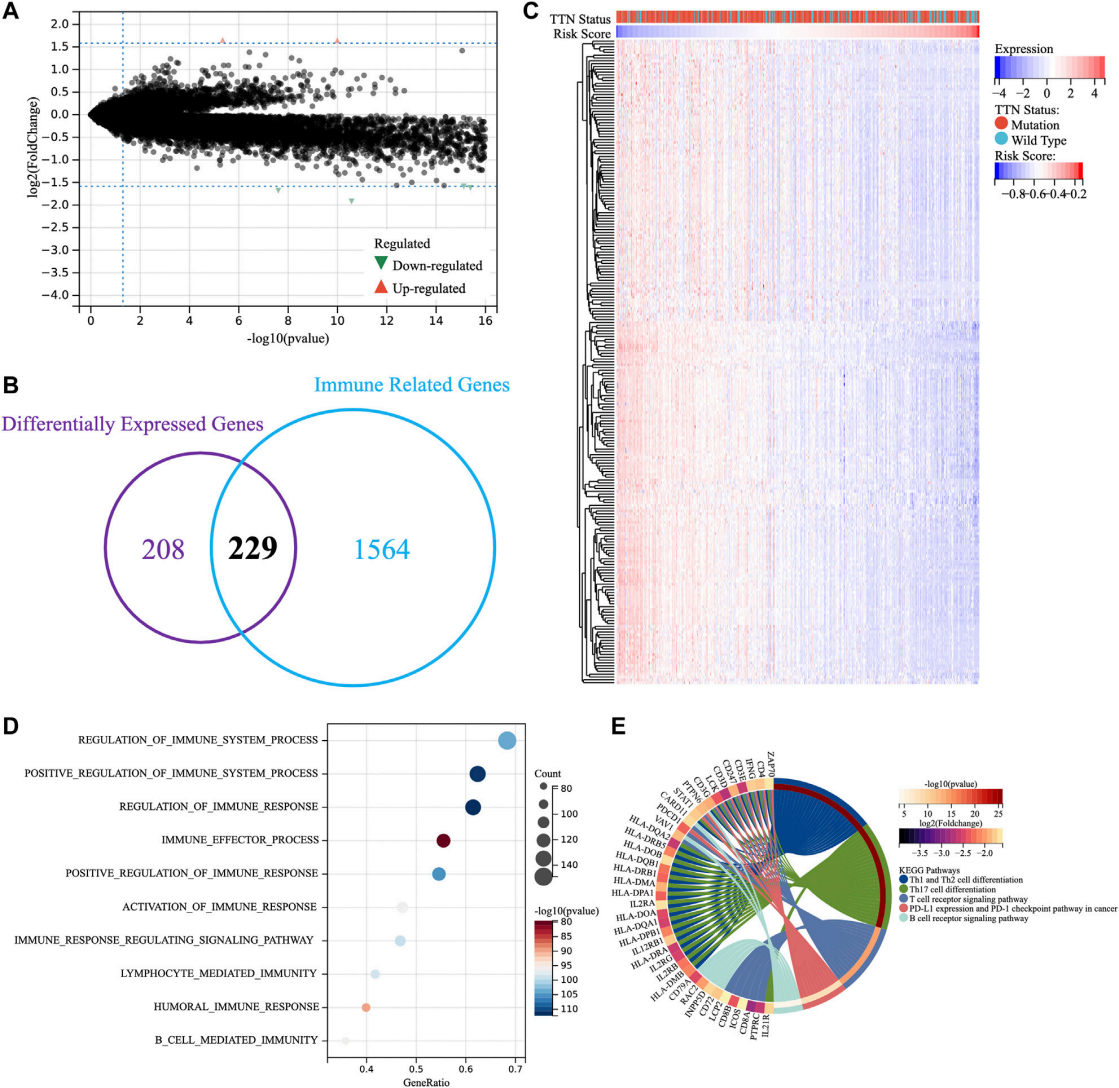
Enrichment analysis of TM-IPM. **(A)** Differentially expressed genes between high-risk and low-risk groups. **(B)** Overlapping differentially expressed genes and immune-related genes. **(C)** Heatmap of differentially expressed immune-related genes. **(D)** Gene ontology (GO) analysis of differentially expressed immune-related genes. **(E)** Kyoto Encyclopedia of Genes and Genomes (KEGG) pathway analysis of differentially expressed immune-related genes.

The above results suggested the association of TTN mutation with immune processes and the validity of TM-IPM in predicting the prognosis of SKCM patients, and we next explored the potential functions of TM-IPM. Therefore, we first performed differential expression analysis with the *limma* package for the high- and low-risk groups (|fold change| ≥ 3 and adj. *p*-value <0.05), in which 3 genes were upregulated while 434 genes were downregulated (Figure 4A, Supplementary Table S3). DEGs can clearly distinguish between high-and low-risk SKCM patients (Supplementary Figure S3). To investigate whether immune-related DEGs existed between the two groups, the DEGs and immune-related genes were overlapped (Figure 4B). Figure 4C showed that these immune-related DEGs were highly expressed mainly in the low-risk group. GO and KEGG enrichment analysis were performed based on these immune-related DEGs. As shown in Figure 4D, the top 10 most significantly enriched immunological processes in the low-risk group of tumors were listed. The results of GO (biological process, BP) analysis were significantly correlated with the regulation of many immunological processes, mainly including the regulation of immune system process and immune response. As shown in Figure 4E, KEGG enrichment analysis showed that low-risk SKCMs are regulated by multiple immune-related pathways, mainly including Th1 and Th2 cell differentiation, Th17 cell differentiation, T cell receptor signaling pathway, PD-L1 expression and PD-1 checkpoint pathway in cancer, and B Cell receptor signaling pathway.

## The immune landscape was evaluated among low- and high-risk groups

**FIGURE 5:**
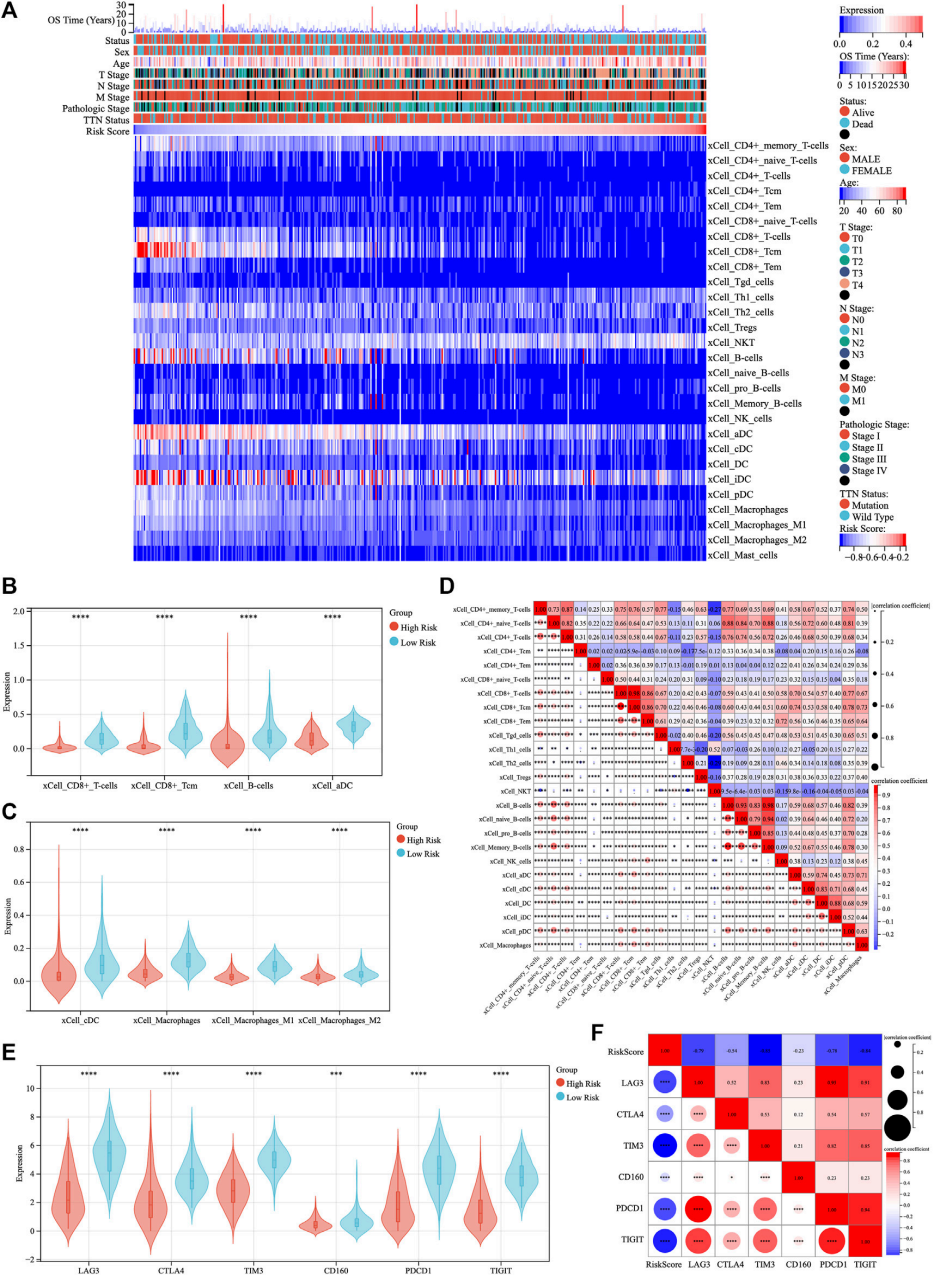
The landscape of immune infiltration in low- and high-risk SKCM patients. **(A)** The heatmap of immune cell infiltration in SKCM was analyzed by xCell method and ranked by risk score. **(B,C)** The violin plot compared the scores of eight infiltrated immune cells between the low- and high-risk groups. **(D)** Correlation between immune cell scores. **(E)** Expression levels of immune checkpoints between low- and high-risk groups. **(F)** Correlation between immune checkpoints and risk scores.

The existence of multiple immune-related biological functions and signaling pathways has been demonstrated in the low-risk group of patients with TM-IPM. Next, we evaluated the differences in immune cell types between low- and high-risk SKCM patients by the xCell, CIBERSORTx and quanTIseq methods. Figure 5A, Supplementary Figures S4A, C demonstrate the trend of infiltration of immune cells in SKCM patients from TCGA database. Significantly different immune cell infiltration profiles exist between different risk groups. To facilitate the observation of differences in immune cells by subtype, we compared immune cell infiltration scores with significant trends between the high- and low-risk groups (Figures 5B,C, Supplementary Figures S4B, D). From the results of the xCell method, the scores of CD8^+^ T cells, CD8^+^ central memory T cells (Tcm), B Cells, activated dendritic cell (aDC), conventional DC (cDC), and macrophages (including M1 and M2 types) were significantly higher in the low-risk group compared with the high-risk group. Additionally, different subpopulations of tumor-infiltrating immune cells showed different degrees of correlation with each other. Moreover, the results of CIBERSORTx showed that CD8^+^ T cell scores were significantly higher in the low-risk group than in the high-risk group; conversely, macrophages (M0 and M2) and mast cells scored higher in the high-risk group than in the low-risk group (Figure S4B). Further, the results of quanTIseq showed that the infiltration levels of CD8^+^ T cells and macrophages (M1 and M2) were significantly higher in the low-risk group than in the high-risk group, while the levels of DCs were higher in the high-risk group than in the low-risk group (Figure S4D). Combining these results, we hypothesize that CD8^+^ T cells may be the predominantly infiltrating immune cells in the low-risk group of SKCM and play an essential role. Finally, ESTIMATE was used to assess the correlation between the risk score of TM-IPM and the immune microenvironment. The risk score was significantly negatively correlated with ESTIMATEScore, ImmuneScore and StromalScore (Figure S5A). Based on the results of immune cell infiltration, we further explored the differences in genes associated with T cell function between high- and low-risk groups. The results showed that interleukin (IL) 2, IL7, IL15 and Interferon-γ (IFN-γ) were significantly higher in the low-risk group than in the high-risk group (Supplementary Figure S5B).

### Differences in immune checkpoint genes between low- and high-risk groups

The expression of six immune checkpoint genes (PDCD1, TIGIT, CD160, TIM3, CTLA4, and LAG3) was significantly higher in the low-risk patients than in the high-risk patients (Figure 5E). There was a significant negative correlation between risk scores and the expression levels of immune checkpoint genes, while there was a moderate to high correlation among immune checkpoint genes (Figure 5F).

### The TM-IPM is independent of conventional clinical characteristics

**FIGURE 6:**
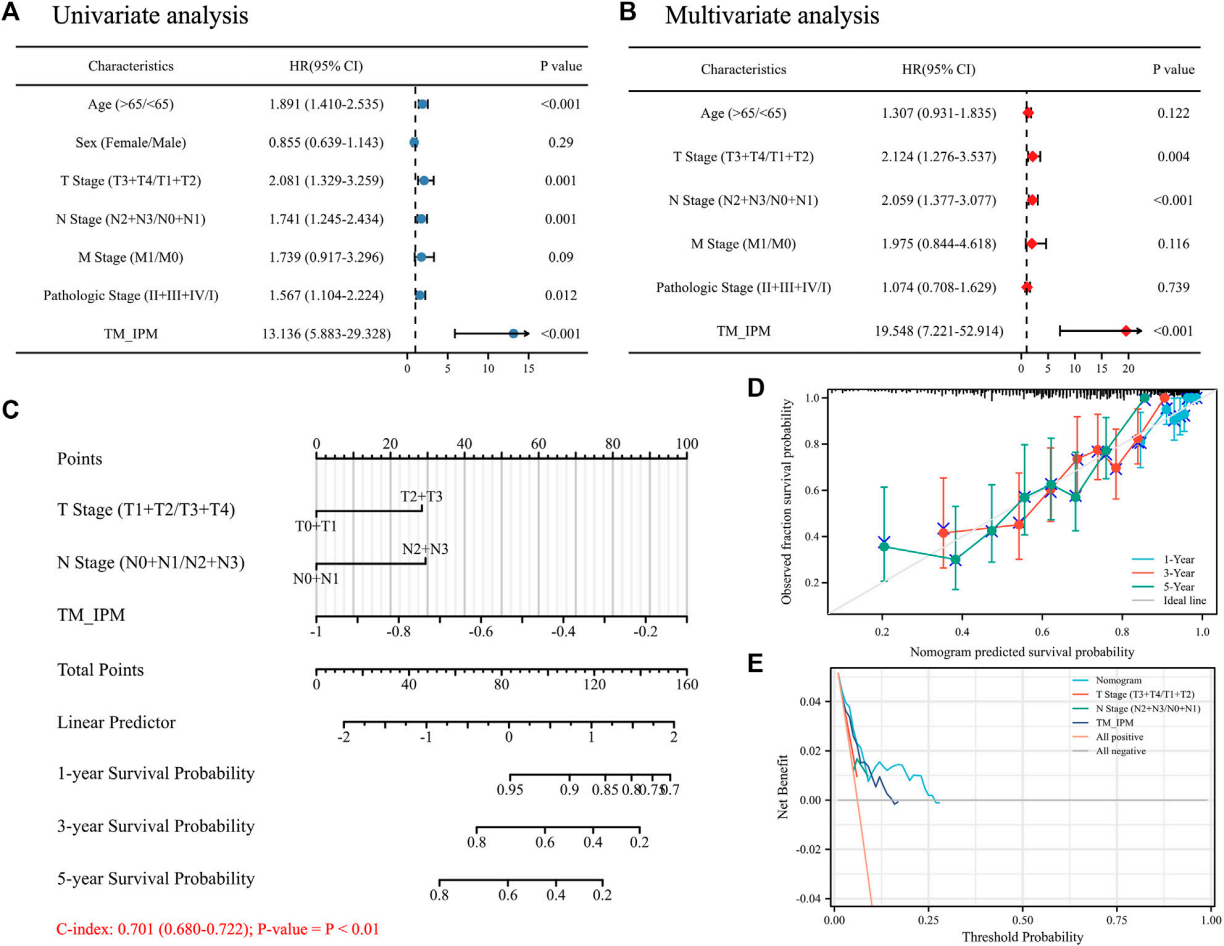
The relationship between TM-IMP and other clinical characteristics. **(A)** Univariate regression analysis of TM-IMP with other clinical characteristics (age, sex, T/N/M stage, and Pathologic stage). **(B)** Multivariate regression analysis of TM-IMP with other clinical characteristics (age, T/N/M stage, and Pathologic stage). **(C)** Nomogram was constructed based on the results of multifactorial regression analysis, which predicted the probability of 1-, 3-, and 5-year overall survival (OS) of SKCM patients. **(D)** Calibration plot of the nomogram predicting the probability of OS at 1, 3 and 5 years. **(E)** Plots of decision curve analysis for the nomogram.

Univariate and multivariate Cox regression analyses were performed to investigate whether the prognostic value of TM-IPM was independent of other clinical factors in the TCGA SKCM cohort. Age, T/N stage, pathological stage, and risk score of TM-IPM were independent risk factors (Figure 6A). Taking into account clinical characteristics, such as age, T/N stage, and pathological stage, TM-IPM remained an independent prognostic factor, thus demonstrating its robustness in independently predicting SKCM prognosis (Figure 6B). The risk of death increased with increasing risk score of TM-IPM (HR = 13.136, 95% CI = 5.883–29.328). A similar, multivariate Cox regression analysis showed that TM-IPM was significantly associated with survival information (*p* < 0.001) and risk score (HR = 19.548, 95% CI = 7.221–52.914).

### Construction and validation of a nomogram based on the TM-IPM

Based on the results of multivariate Cox regression analysis, we constructed a nomogram integrating independent clinical risk factors (T\N stage) and TM-IPM, which may improve the quantitative approach for clinicians to predict the prognosis of SKCM patients (Figure 6C). This nomogram can predict OS of 1, 3, and 5 years in the TCGA SKCM cohort. For OS prediction, the C-index of the nomogram was 0.701 (0.680–0.722) (*p* < 0.01). Additionally, the calibration curve suggested that its prediction was accurate (Figure 6D). Furthermore, DCA results showed that our nomogram has a high degree of clinical utility (Figure 6E).

### Chemotherapy drug sensitivity analysis

**FIGURE 7:**
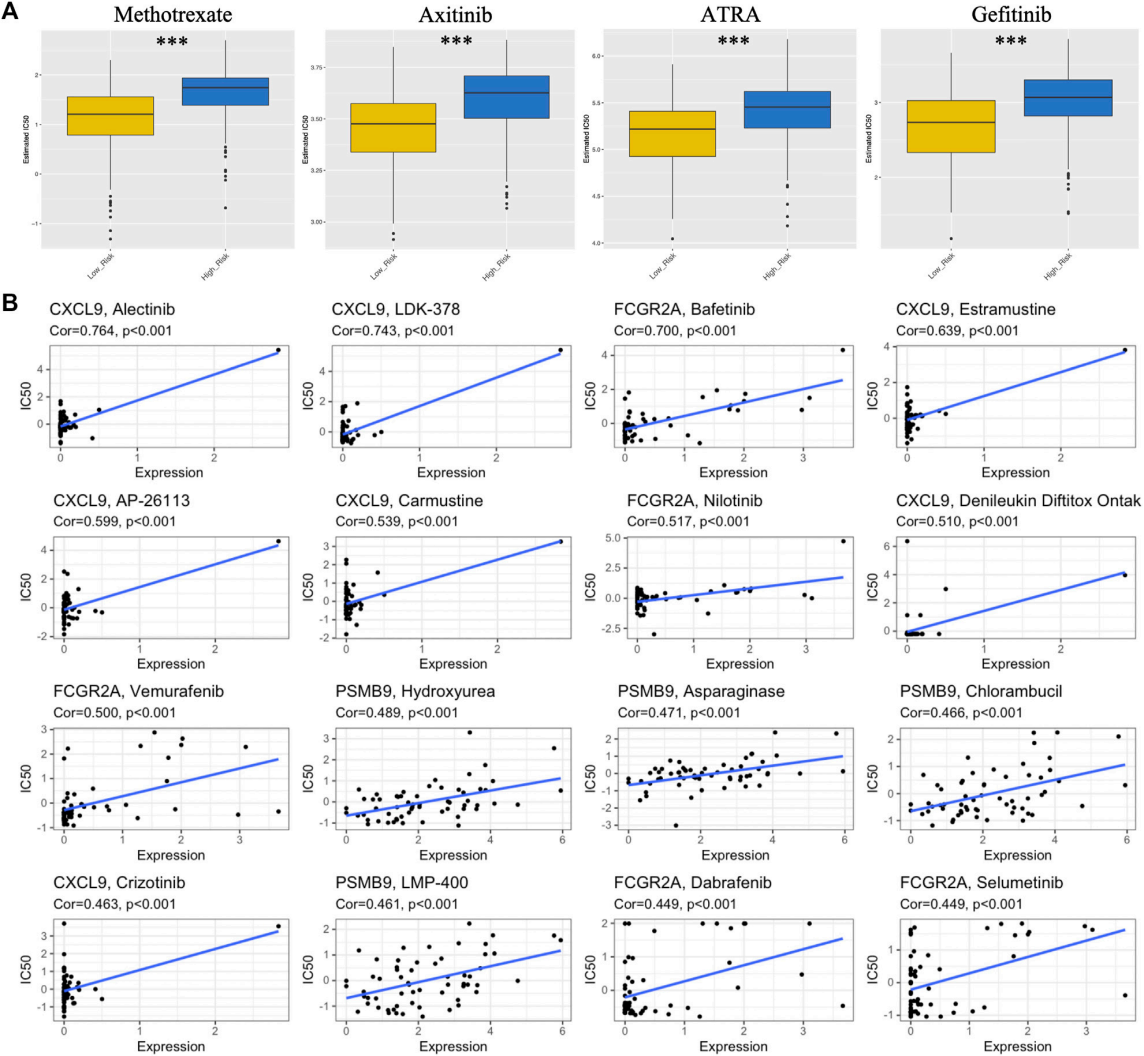
Association between risk score, TM-IPM genes, and chemosensitivity in SKCM. **(A)** Box plots of estimated IC50 for ATRA, gefitinib, axitinib, and methotrexate in high- and low-risk groups. **(B)** Scatter plot of the relationship between the expression of TM-IPM genes and drug sensitivity.

As shown in Figure 7A, an analysis was performed on the sensitivity of four types of chemotherapy drugs: ATRA, gefitinib, axitinib and methotrexate. The IC50 values for ATRA, gefitinib, axitinib, and methotrexate were significantly lower in the low-risk group than in the high-risk group, suggesting that patients with lower risk scores were more sensitive to these chemotherapeutic drugs. In addition, the expression of four IRGs in NCI-60 cell lines was investigated, while revealing the relationship between their expression levels and drug sensitivity. The results showed that these four IRGs correlated with some chemotherapeutic drug sensitivities (*p* < 0.001, Figure 7B; Supplementary Table S4).

## Discussion

Recent studies have shown that TTN is the most frequently mutated gene in tumors (Oh et al., 2020). Consistent with this view, TTN possesses a long exon length in the entire genome, which results in a greater number of mutant loci (Jia et al., 2019). Studies have shown that higher mutation rates are significantly associated with TMB, which may contribute to the generation of new antigens and stimulate T cell recognition, ultimately affecting the efficacy of immunotherapy for patients (Oh et al., 2020; Jardim et al., 2021). Jia et al. found a significant positive correlation between objective remission rates and TTN mutation frequency in melanoma patients treated with anti-PD-1/PD-L1/CTLA-4 monotherapy (Jia et al., 2019). Therefore, it is essential to further investigate the immune-related effects of TTN status. However, it is still unknown how TTN mutation affects the immunophenotypic and prognosis of SKCM. Further, it is important to develop validated immune-related prognostic models to determine the immune status of patients, which may serve as powerful prognostic biomarkers and allow for the classification of patients to improve the effectiveness of immunotherapy. Numerous studies have developed immune-related clinical prediction models (Hu et al., 2020; Ping et al., 2021; Tian et al., 2021); however, they did not associate TTN mutation, the most common mutated gene in SKCM, with immune phenotypic regulation. In this study, we found that TTN mutation was significantly associated with longer OS in SKCM patients compared to wild type. To investigate whether TTN mutation is associated with immunomodulation of SKCM, we performed GSEA to compare the immune processes of SKCM with different TTN status. Furthermore, we obtained immune-related genes affected by TTN mutation based on GSEA results and established a four-gene TM-IPM that can identify high-risk SKCM patients with poor prognosis.

IFN-γ has the function of activating cellular immunity and subsequently stimulating anti-tumor immune responses, which promotes apoptosis and inhibits the proliferation of tumor cells (Jorgovanovic et al., 2020). CXCL9 is a chemokine induced by IFN-γ. The CXCL9/10/11/CXCR3 axis is capable of regulating immune cell migration, differentiation and activation. It has been shown that CXCL9 can inhibit tumor growth by mediating lymphocyte infiltration to the lesion site (Tokunaga et al., 2018). Elevated CXCL9 can suppress melanoma growth by regulating effector T cells, thereby promoting anti-tumor adaptive immunity (Xiao et al., 2018). PSMB8 is a subunit of the immunoproteasome that degrades cellular proteins to produce peptides for antigen presentation, which induces an effective immune response in CD8^+^ T cells (Kalaora et al., 2020; Tripathi et al., 2021). PSMB8 deficiency has been shown to promote tumor growth in a mouse model of melanoma (Leister et al., 2021). CD274, also known as PD-L1, can bind to PDCD1 to downregulate T cell function in melanoma (Eggermont et al., 2014). It was found that IFN-γ-mediated CD274 upregulation and increased OS in metastatic melanoma were both significantly associated with CD8^+^ T cells (Vaxevanis et al., 2022). Furthermore, patients with lower CD274 levels had reduced T cell infiltration and responded poorly to treatment with immune checkpoint inhibitors (Vaxevanis et al., 2022). The *FCGR2A* gene, a member of the gene family encoding the immunoglobulin Fc receptor, regulates antibody-dependent cytotoxicities, which are essential for the elimination of cancer cells (Dai et al., 2021). FCGR2A was found to be positively associated with survival in melanoma patients (Zhong et al., 2022). In our study, CXCL9, PSMB9, CD274, and FCGR2A were combined for the first time to predict the prognosis of SKCM patients, and they were all associated with a good prognosis of patients.

Moreover, TM-IPM was shown to be an independent predictor of prognosis in SKCM patients. Additionally, in order to more accurately predict patient prognosis, we constructed a nomogram by combining other clinical characteristics (T/N stage) and TM-IPM. The calibration curves showed a good agreement between the observed values and the predicted values of 1-year, 3-year and 5-year OS. This model provided a personalized scoring system for individual tumors compared to traditional methods of assessing patient prognosis (such as T/N/M and pathologic stage), so our nomogram may be a useful tool to assist future clinicians.

In the heterogeneous tumor microenvironment, T cells are critical components of the immune infiltrate. Among them, CD8^+^ cytotoxic T cells have the ability to directly clear tumor cells (Hashimoto et al., 2018). However, for some reason, such as continuous antigen stimulation, these cells enter a state of “dysfunction” or “exhaustion” and express immune checkpoint proteins that help tumor cells escape immune clearance (Li et al., 2019). Moreover, checkpoint blockade therapies targeting tumor-specific T cells have greatly improved the outcome of cancer treatment (Ribas and Wolchok, 2018). Furthermore, infiltrating CD8^+^ cytotoxic T cells were positively associated with longer disease-free survival (DFS) and/or improved OS in melanoma patients (Bruni et al., 2020). In our study, the infiltration level of CD8^+^ T cells in SKCM was significantly higher in the low-risk group than in the high-risk group. Elevated expression of immune checkpoints (PDCD1, TIGIT, CD160, TIM3, CTLA4, and LAG3) may indicate that T cell function is being reduced; however, IFN-γ expression was significantly higher in the low-risk group than in the high-risk group, suggesting that CD8^+^ T cells in SKCM in the low-risk group had not yet entered a stage of severe exhaustion (McLane et al., 2019). In addition, we compared the cytokines IL-7 and IL-15 expression levels between the two groups, which may represent a relatively stable state of memory T cells in the low-risk group (McLane et al., 2019). Interestingly, this result is consistent with the KEGG analysis, with significant enrichment of Th1, Th2, and Th17 cell differentiation in SKCM in the low-risk group compared to the high-risk group, possibly associated with high levels of IL2 expression. The loss of IL-2 production occurred early in T cell depletion, and IL2 expression was higher in the low-risk group than in the high-risk group, which also represented a residual cytotoxicity and tumor cell clearance function of CD8^+^ T cells in the low-risk patients.

In addition, we performed a prediction of response to chemotherapeutic drugs and analysis of four genes of TM-IPM for correlation with drugs in low- and high-risk groups. The results showed that patients in the low-risk group were more sensitive to chemotherapeutic drugs compared to the high-risk group and that the four genes of TM-IPM were significantly positively correlated in responsiveness to drugs, which may explain the longer survival in the low-risk group. Our study provides new perspectives on the SKCM immune microenvironment and immune-related therapies. Nevertheless, our study is limited because it is based on public databases. Furthermore, functional and mechanistic studies of these four genes should be performed individually or in combination to demonstrate the availability and scientific validity of TM-IPM.

## Conclusion

TTN has the highest mutation rate in SKCM and thus it plays a crucial role in tumorigenesis and progression. The survival of SKCM patients with TTN mutation was significantly longer than that of wild-type patients. Additionally, TTN mutation was markedly associated with four immune-related biological processes by GSEA. Based on this, we identified and validated for the first time a TM-IPM based on four immune-related genes (CXCL9, PSMB9, CD274 and FCGR2A) with independent prognostic significance for SKCM patients and reflecting the immune characteristics of the SKCM microenvironment. Importantly, this research was the first to construct a clinical prognostic model associated with TTN mutation, which can be used as a reference for other cancers that also have high TTN mutation rates. In this study, TM-IPM explains the mechanism by which TTN mutation affects the outcome of SKCM patients from an immunological perspective. Collectively, TM-IPM was able to classify SKCM patients into high- and low-risk groups. SKCM patients in the low-risk group had a better prognosis compared to those in the high-risk group. Moreover, the low-risk group of SKCM patients was more sensitive to chemotherapeutic drugs. Therefore, TM-IPM was a useful tool to assist clinicians in predicting the prognosis and response to chemotherapy in patients with SKCM.

## Data Availability

The original contributions presented in the study are included in the article/Supplementary Material, further inquiries can be directed to the corresponding author.
