# Supplementary material for: Identification and validation of a TTN-associated immune prognostic model for skin cutaneous melanoma
Source: Front Genet. 2023 Jan 10;13:1084937. doi: 10.3389/fgene.2022.1084937 (PMC9871619; doi:10.3389/fgene.2022.1084937)
Supplement: Supplementary file 1 [file DataSheet1.zip › Supplementary Materials/Supplementary Figures.docx]

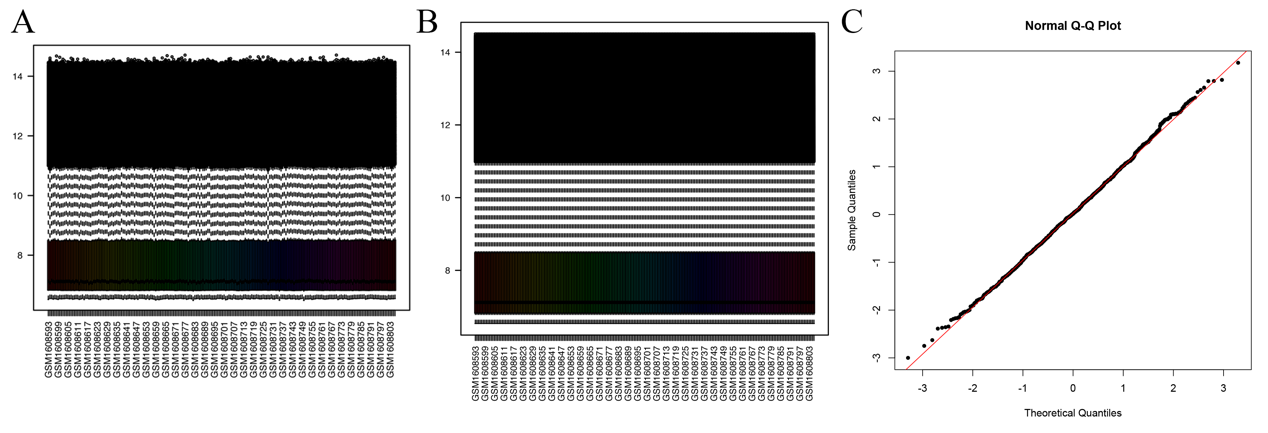


Figure S1: (A) Box plot of GSE65904 data before normalization. (B) Box plot of GSE65904 data after normalization. (C) Q-Q plot of GSE65904 data after normalization.


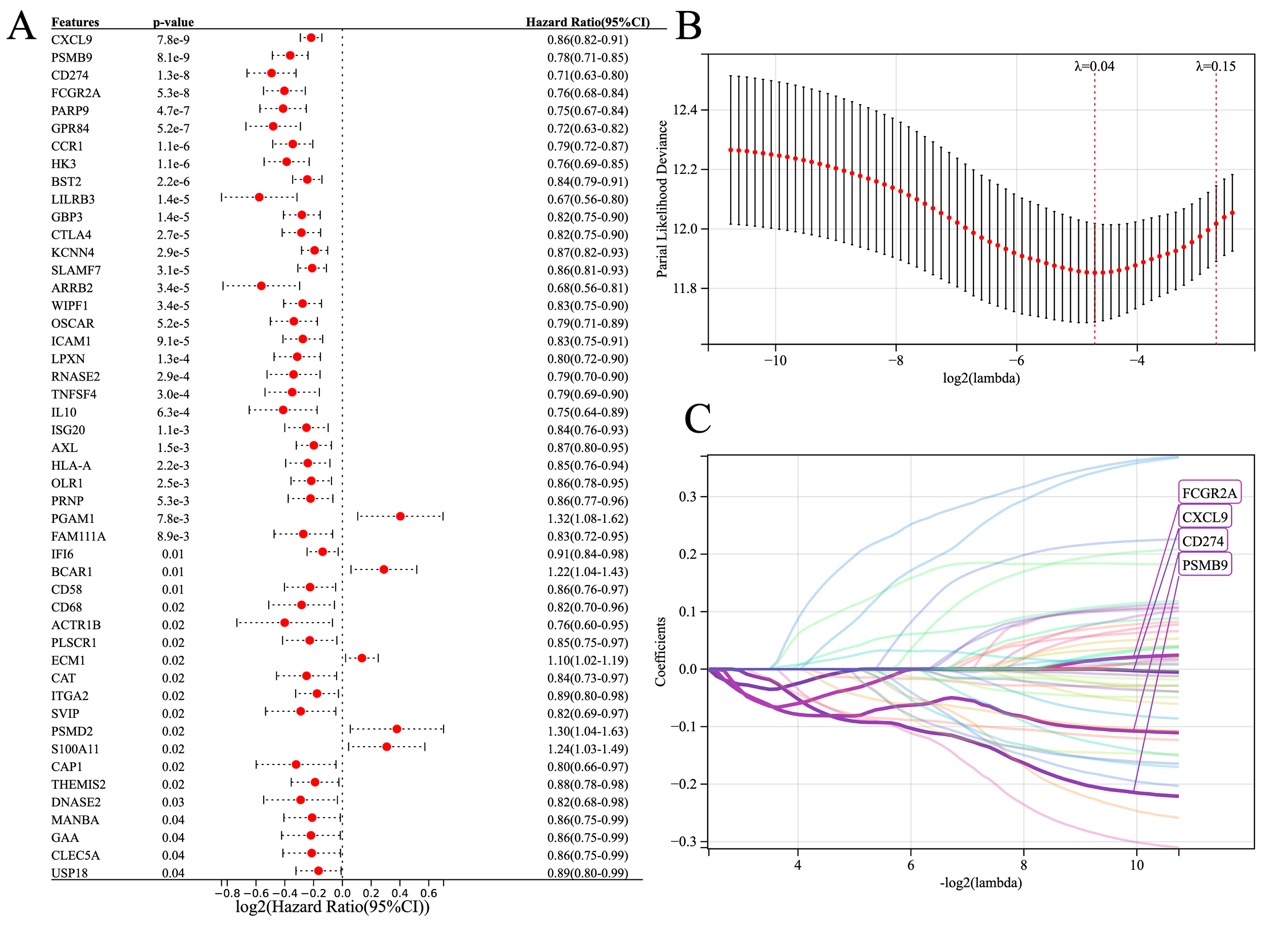


Figure S2: (A) Univariate regression analysis of 48 differentially expressed immune-related genes. (B) Screening of immune-related genes for the construction of TM-IPM by LASSO regression analysis. Two vertical dashed lines were plotted according to the minimum criterion (left) and at the optimal value (right). (C) Distribution of LASSO coefficients for the four immune-related genes. Four genes with non-zero coefficients were labeled.


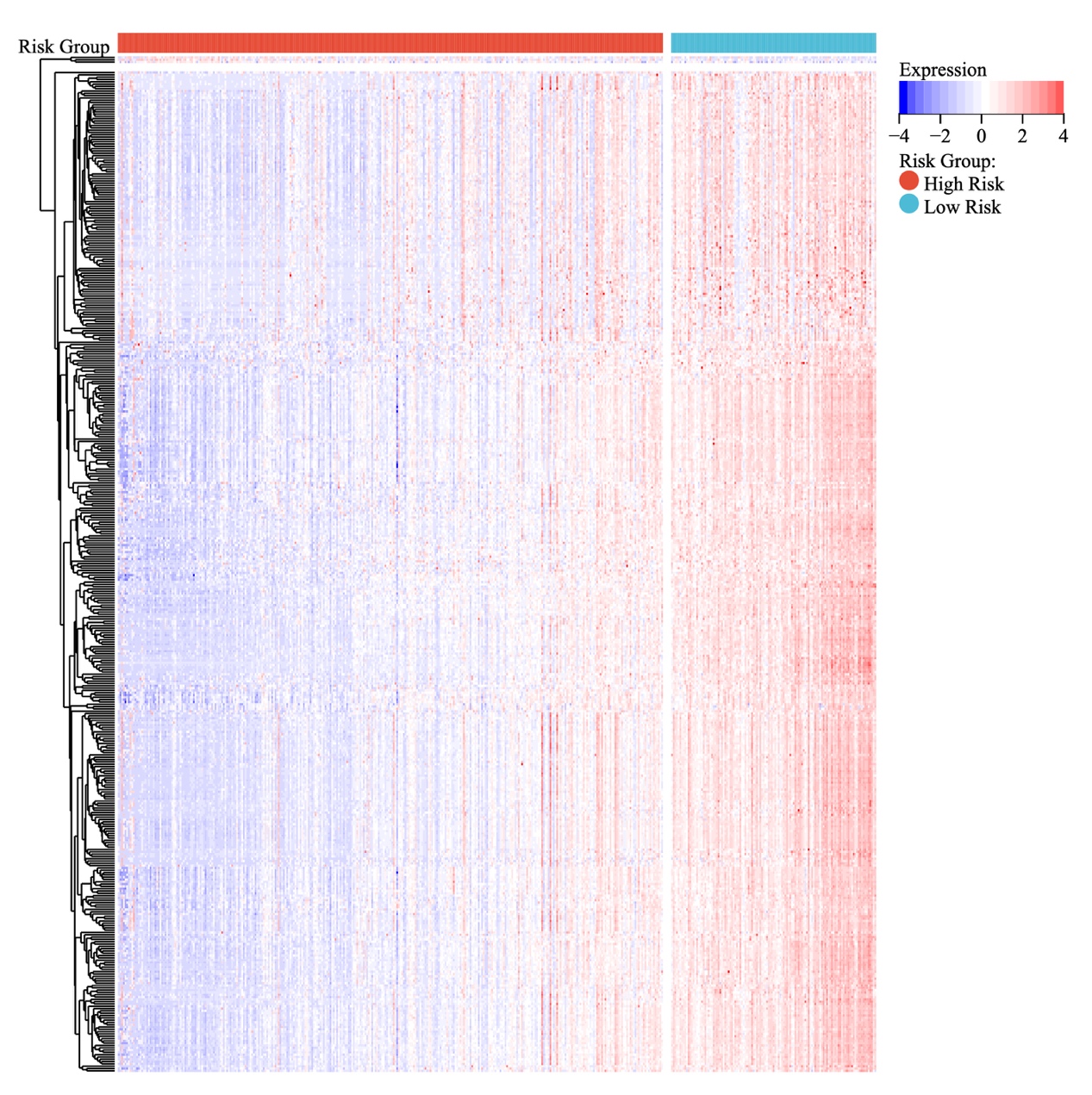


Figure S3: The heatmap of differentially expressed genes between high- and low-risk groups.


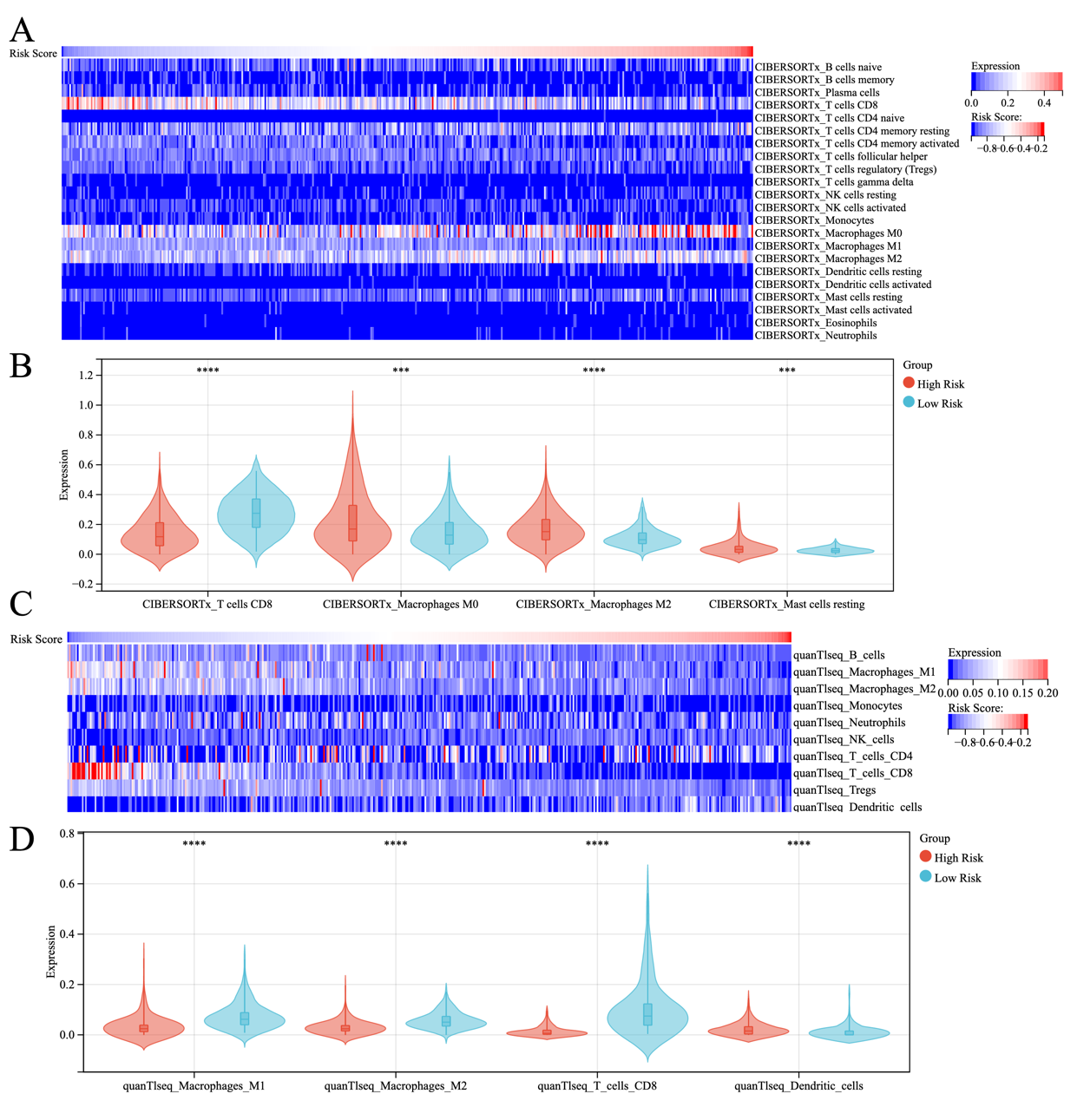


Figure S4: (A) The heatmap of immune cell infiltration in SKCM was analyzed by CIBERSORTx method and ranked by risk score. (B) The violin plot compared the scores of four infiltrated immune cells between the low- and high-risk groups. (C) The heatmap of immune cell infiltration in SKCM was analyzed by quanTIseq method and ranked by risk score. (D) The violin plot compared the scores of four infiltrated immune cells between the low- and high-risk groups.


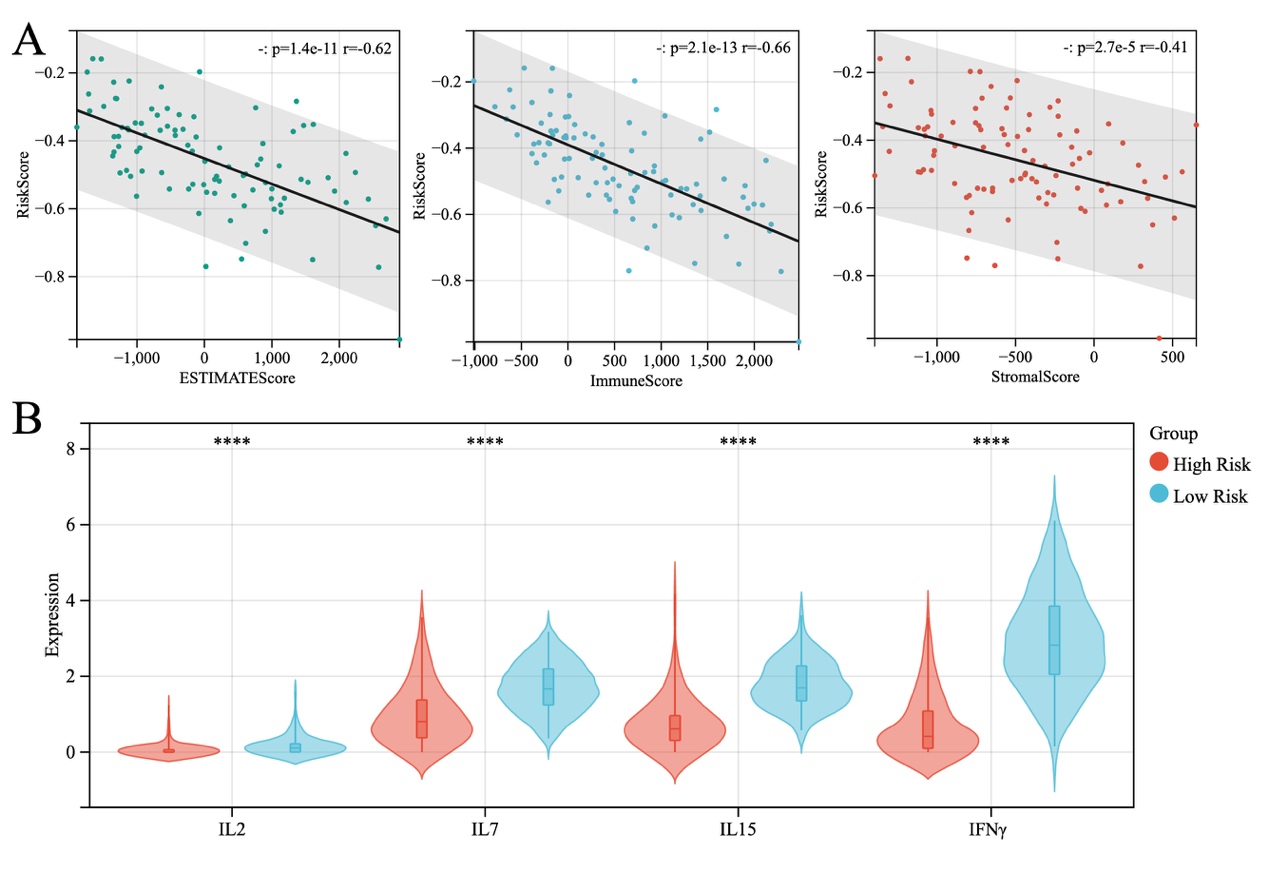


Figure S5: (A) ESTIMATE algorithm was used to assess the immune microenvironment of SKCM. (B) The expression levels of IL2, IL7, IL15 and IFNγ were compared between the low-risk and high-risk groups.
